# Supplementary material for: Causal effects of population dynamics and environmental changes on spatial variability of marine fishes
Source: Nat Commun. 2020 May 26;11:2635. doi: 10.1038/s41467-020-16456-6 (PMC7250893; doi:10.1038/s41467-020-16456-6)
Supplement: Supplementary file 1 — Supplementary Information [file 41467_2020_16456_MOESM1_ESM.pdf]

Supplementary information

Causal effects of population dynamics and environmental changes on spatial variability of marine fishes

Jheng-Yu Wang[1], Ting-Chun Kuo[1,2] and Chih-hao Hsieh[1,3,4,5*]

[1]Institute of Oceanography, National Taiwan University, No.1, Section 4, Roosevelt Road, Taipei 10617, Taiwan

[2]Institute of Marine Affairs and Resources Management, National Taiwan Ocean University, No.2, Peining Road, Jhongjheng District, Keelung City 20224, Taiwan

[3]Institute of Ecology and Evolutionary Biology, Department of Life Science, National Taiwan University, No.1, Section 4, Roosevelt Road, Taipei 10617, Taiwan

[4]Research Center for Environmental Changes, Academia Sinica, No.128, Section 2, Academia Road, Nankang, Taipei 11529, Taiwan

[5]National Center for Theoretical Sciences, No.1, Section 4, Roosevelt Road, Taipei 10617, Taiwan

[*]Corresponding author; Email: chsieh@ntu.edu.tw

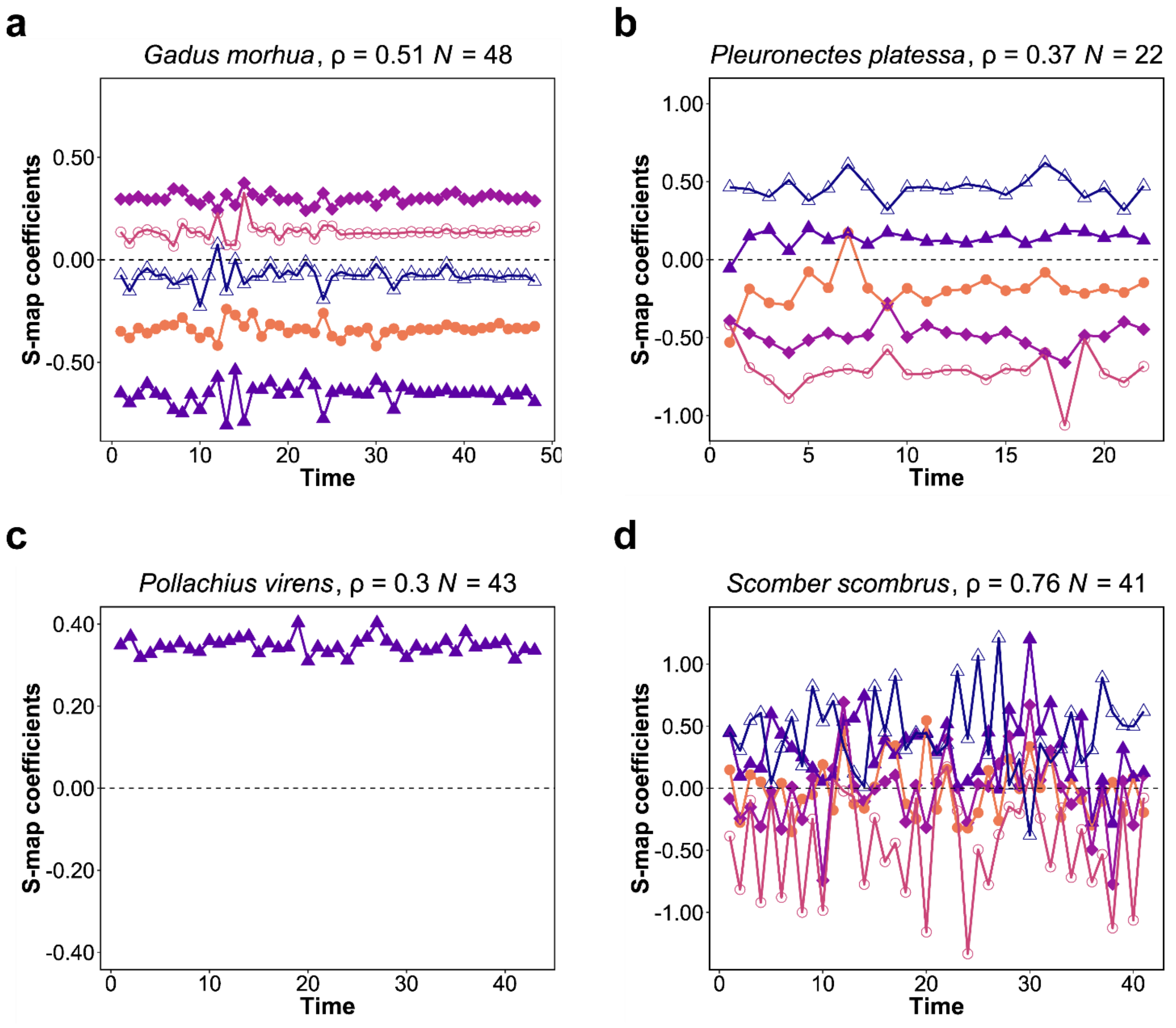

Supplementary Figure 1 | Time series of over-time influential strengths of selected causal variables on population spatial variability. Influential strengths were coefficients of S-map model estimated at each time step (see Methods). A positive S-map coefficient indicates a positive causal effect of the variable on population spatial variability, and vice versa. Magnitude of S-map coefficient represents strength of causal effects. ρ indicates performance of S-map model. Significant S-map results were detected for (**a**) Atlantic cod (**b**) plaice (**c**) saithe, and (**d**) Atlantic mackerel (p-value < 0.1, one-sided Student's t-test on ρ). Causal variables were selected according to embedding dimension and their rank of causal effects determined by convergence cross mapping (CCM; see Table 1). Each variable is labeled as follows: age diversity: orange circle; abundance: red hollow circle; Atlantic Multidecadal Oscillation: light purple square; temperature: dark purple triangle; spatial variability of temperature: dark blue hollow triangle.

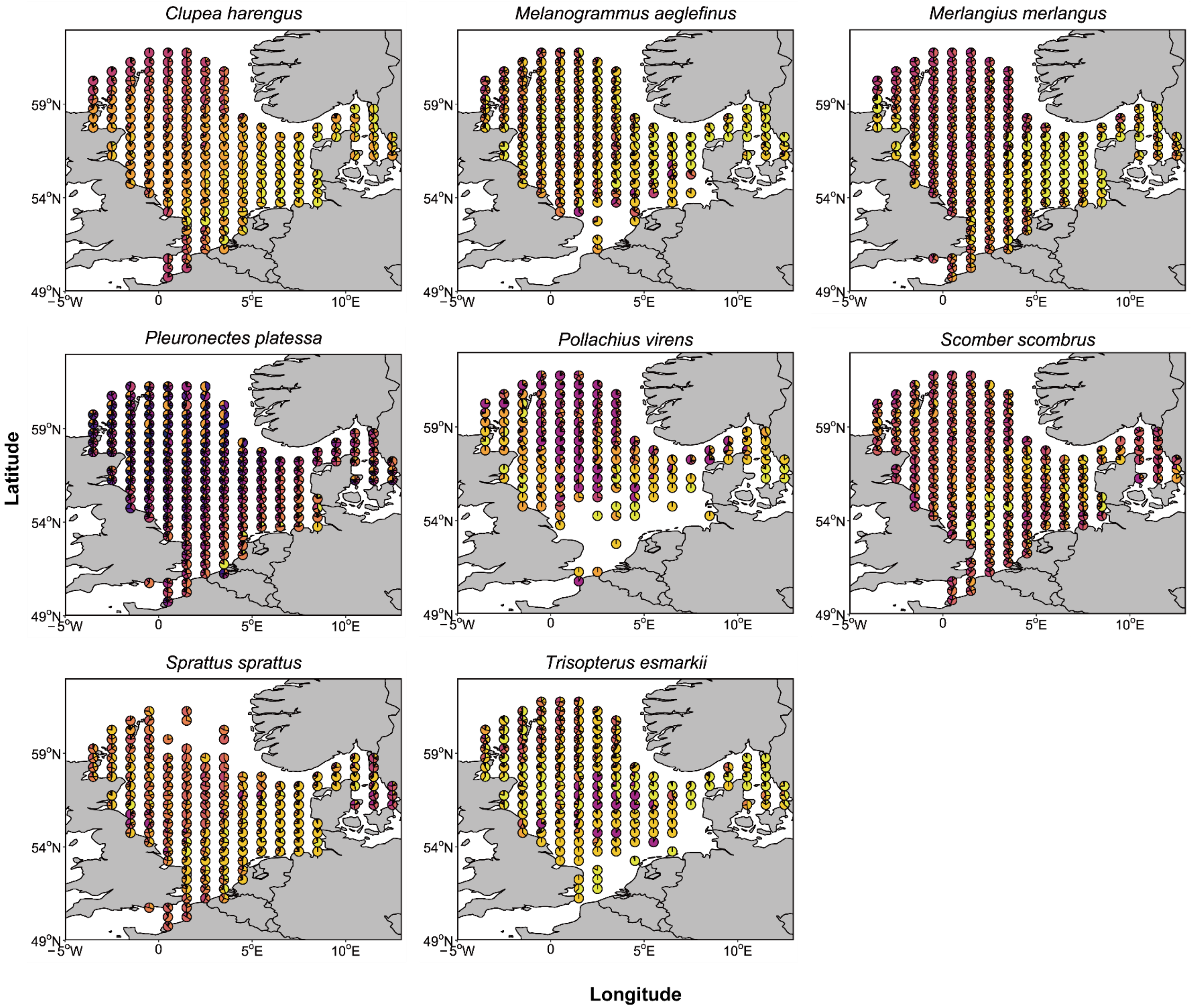

Supplementary Figure 2 | Proportion of abundance of each age class in various survey locations for all study species except for cod (*Gadus morhua*). The spatial pattern was computed by averaging abundance of each age class during the study period. Younger age classes were marked as lighter colors. The figure indicated that different age classes lived in different habitats. Distribution of each age class for cod was shown in Figure 3 in the main text as an example. (Note that this figure is better viewed in the electronic version).

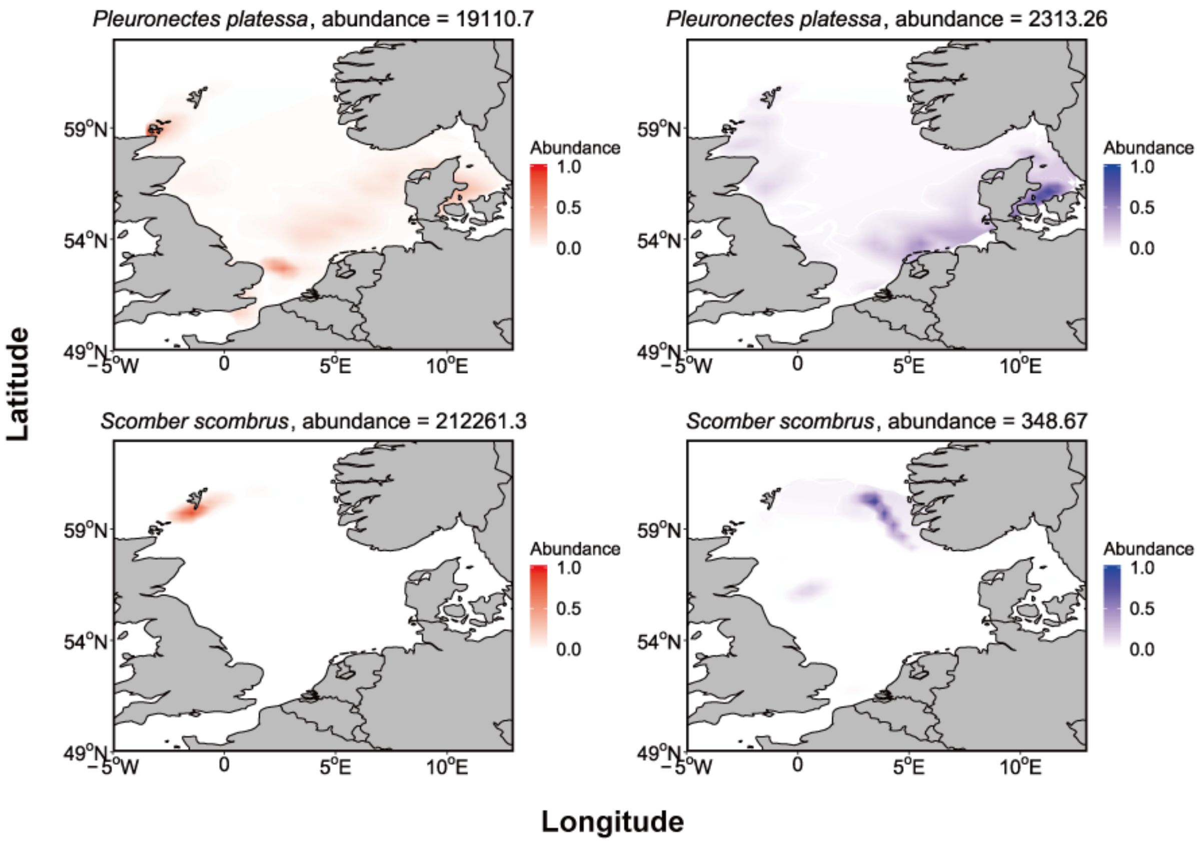

Supplementary Figure 3 | Population spatial distribution at the time when abundance was highest (left) versus lowest (right) for plaice and Atlantic mackerel. Populations tended to distribute more evenly in space when their abundance was higher. Our S-map result also indicated a negative abundance effect on spatial variability for plaice and Atlantic mackerel.

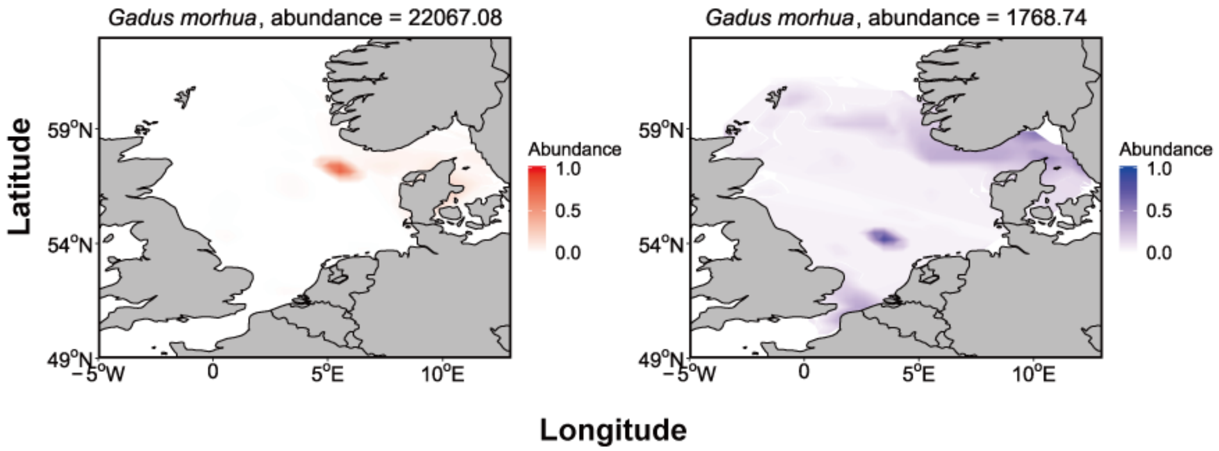

Supplementary Figure 4 | Population spatial distribution at the time when abundance was highest (left) versus lowest (right) for Atlantic cod. Populations tended to distribute more evenly in space when their abundance was lower. Our S-map result also indicated a positive abundance effect on spatial variability for Atlantic cod.

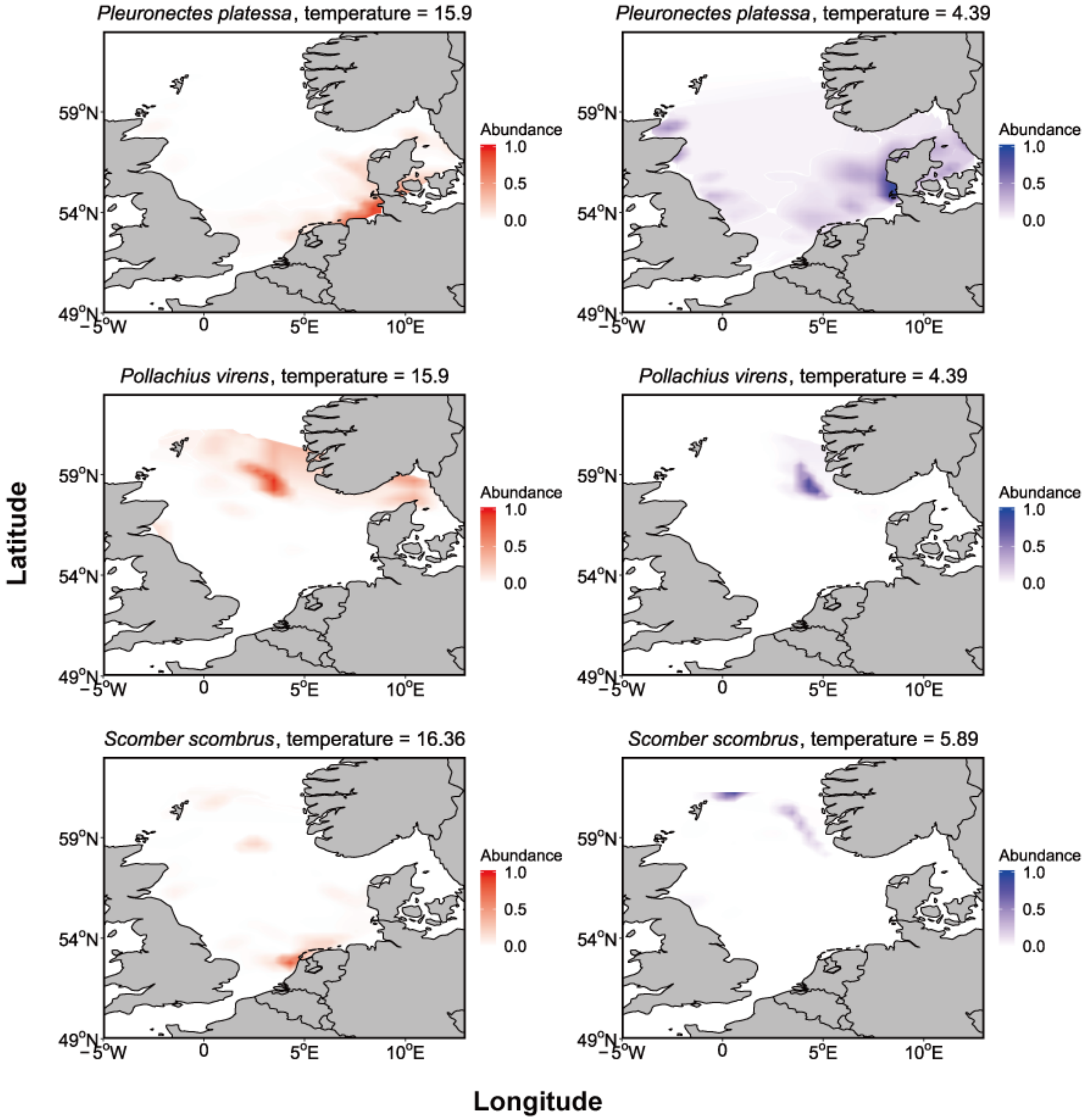

Supplementary Figure 5 | Population spatial distribution at the time when temperature was highest (left) versus lowest (right) for plaice, saithe and Atlantic mackerel. Populations tended to distribute more evenly in space when temperature was lower. Our S-map result also indicated a positive temperature effect on spatial variability for plaice, saithe and Atlantic mackerel.

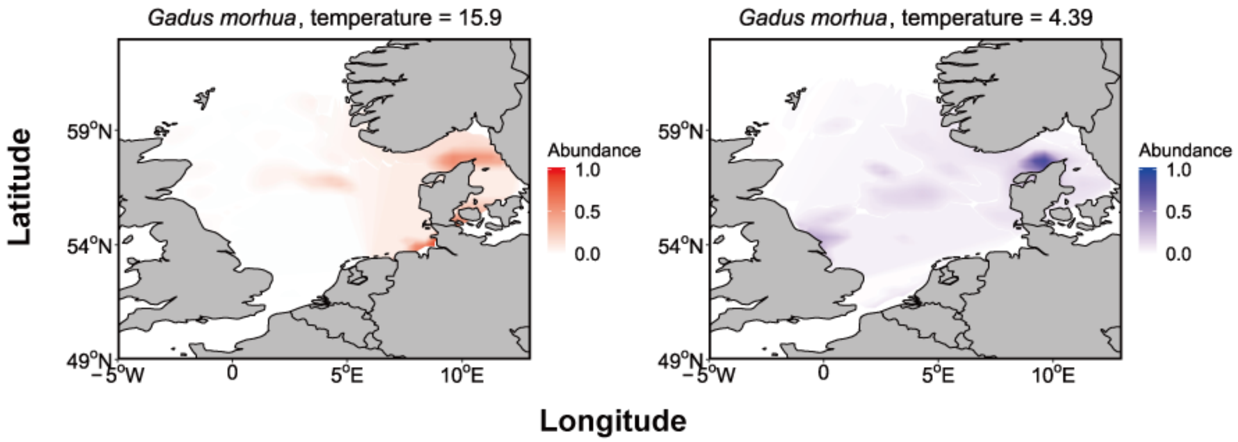

Supplementary Figure 6 | Population spatial distribution at the time when temperature was highest (left) versus lowest (right) for Atlantic cod. Populations tended to distribute more evenly in space when temperature was higher. Our S-map result also indicated a negative temperature effect on spatial variability for Atlantic cod.

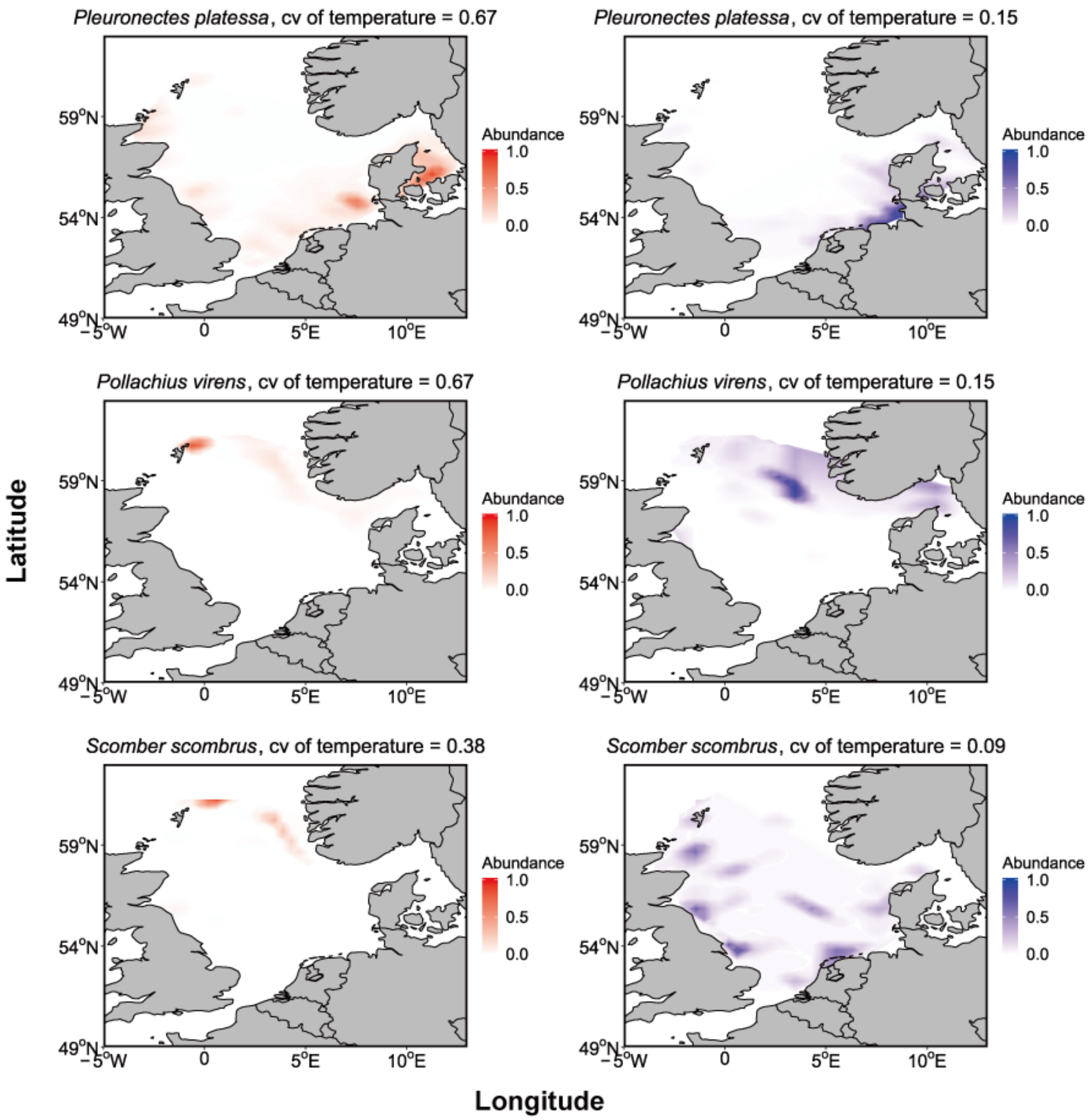

Supplementary Figure 7 | Population spatial distribution at the time when spatial variability of temperature was highest (left) versus lowest (right) for plaice, saithe and Atlantic mackerel. Populations tended to distribute more evenly in space when spatial variability of temperature was lower. Our S-map result also indicated a positive causal effect of spatial variability of temperature on spatial variability for plaice, saithe and Atlantic mackerel. CV, coefficient of variation.

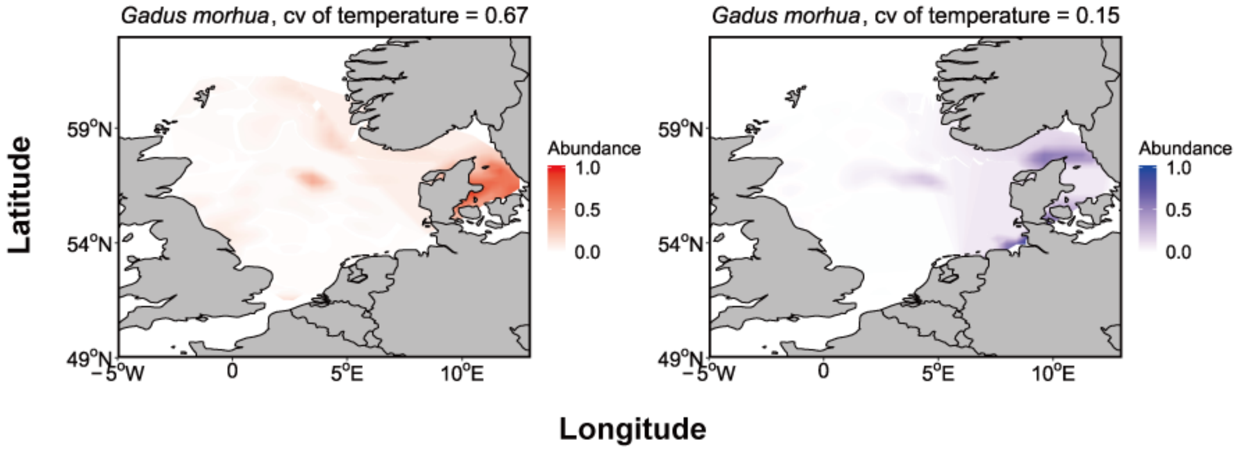

Supplementary Figure 8 | Population spatial distribution at the time when spatial variability of temperature was highest (left) versus lowest (right) for Atlantic cod. Populations tended to distribute more evenly in space when spatial variability of temperature was higher. Our S-map result also indicated a negative causal effect of spatial variability of temperature on spatial variability for Atlantic cod. CV, coefficient of variation.

Supplementary Table 1 │ Biological information for study species.

Species	Common name	Life style	Biogeography[1]	*b*
Clupea harengus	Atlantic herring	Benthopelagic	Boreal	2.11
Gadus morhua	Atlantic cod	Benthopelagic	Boreal	2.56
Melanogrammus aeglefinus	Haddock	Demersal	Boreal	1.62
Merlangius merlangus	Whiting	Benthopelagic	Lusitanian	1.96
Pleuronectes platessa	Plaice	Demersal	Boreal	1.70
Pollachius virens	Saithe	Demersal	Boreal	2.15
Scomber scombrus	Atlantic mackerel	Pelagic	Atlantic	1.85
Sprattus sprattus	Sprat	Pelagic	Lusitanian	2.29
Trisopterus esmarkii	Norway pout	Benthopelagic	Boreal	2.04

b, Taylor's exponent.

Supplementary Table 2 │ Average over-time influential strengths of selected causal variables on population spatial variability.

Species	Library variable: CV of CPUE					*θ*	*ρ*	*p*-value
	**Age diversity**	**Abundance**	**AMO**	**Temperature**	**CV of temperature**			
Clupea harengus (Atlantic herring)	0.1735	-0.1109	-0.2195		0.1934	0	0.0019	0.4951
Gadus morhua (Atlantic cod)	**-0.3372**	**0.137**	**0.299**	**-0.6565**	**-0.0796**	**0**	**0.51**	**< 0.001**
Melanogrammus aeglefinus (haddock)	0.153	0.1156	0.108		-0.4019	3	-0.0664	0.3361
Merlangius merlangus (whiting)	-0.508	-0.6863	0.3198			8	-0.0818	0.2967
Pleuronectes platessa (plaice)	**-0.1928**	**-0.713**	**-0.486**	**0.134**	**0.4592**	**0**	**0.3705**	**0.0448**
Pollachius virens (saithe)				**0.347**		**0**	**0.3031**	**0.0241**
Scomber scombrus (Atlantic mackerel)	**-0.0114**	**-0.4519**	**-0.0332**	**0.3111**	**0.4501**	**3**	**0.7568**	**< 0.001**
Trisopterus esmarkii (Norway pout)	0.2157	-0.0506				6	0.0041	0.4893

CV, coefficient of variation; CPUE, catch per unit effort; AMO, Atlantic Multidecadal Oscillation; *θ*, nonlinearity of dynamical system; *ρ*, performance of S-map model.

Influential strengths were estimated by S-map model at each time step during study period (see Methods), and were averaged over time. Causal variables were selected according to embedding dimension and their rank of causal effects determined by convergence cross mapping (CCM; see Table 1). Significant S-map results are indicated in bold (*p*-value < 0.1, one-sided Student's *t*-test on *ρ*).

Supplementary Table 3 | Average over-time influential strengths of selected causal variables on population spatial variability under alternative combinations of causal variables.

Species	Library variable: CV of CPUE					θ	ρ	p-value
	Age diversity	Abundance	AMO	Temperature	CV of temperature			
Pollachius virens (saithe)					**0.605**	**3**	**0.3328**	**0.0136**
		-0.0228				0	0.0361	0.4081
			-0.0722			0	0.1232	0.202
	0.2017					6	0.1766	0.1176
Trisopterus esmarkii (Norway pout)	0.2157	-0.0506				6	0.0041	0.4893
	0.0613				0.2524	8	-0.0468	0.3801
	0.2274			-0.3363		3	-0.061	0.3453
	0.716		0.2142			8	0.029	0.4278
		-0.309			0.5327	8	-0.1377	0.1836
		-0.3669		0.2471		4	-0.1264	0.204
		-0.1863	-0.8727			8	-0.0909	0.2834
				2.3819	2.6291	8	0.0911	0.2712
			-1.2218		0.4191	8	0.0279	0.4304
			-1.683	-0.9213		8	0.0485	0.3802

CV, coefficient of variation; CPUE, catch per unit effort; AMO, Atlantic Multidecadal Oscillation; θ, nonlinearity of dynamical system; ρ, performance of S-map model.

Influential strengths were estimated by S-map model at each time step during study period (see Methods), and were averaged over time. Significant S-map results are indicated in bold (p-value < 0.1, one-sided Student's t-test on ρ).

Supplementary Table 4 │ Causal effect of examined variables, including fishing mortality, on population age diversity.

Species	Dimensionality (E^*)	Library variable: age diversity					
		F	CV of CPUE	Abundance	AMO	Temperature	CV of temperature
Clupea harengus (Atlantic herring)	2	n.s.	0.3834 (2)	0.2478 (0)	0.3322 (3)	0.2125 (3)	0.1803 (0)
Gadus morhua (Atlantic cod)	2	0.6836 (3)	0.5054 (4)	n.s.	0.6377 (1)	n.s.	0.3049 (1)
Melanogrammus aeglefinus (haddock)	2	n.s.	0.1238 (3)	0.3468 (2)	0.4485 (4)	n.s.	0.2927 (1)
Merlangius merlangus (whiting)	6	0.3546 (1)	0.0792 (4)	0.6871 (4)	0.4536 (1)	0.2479 (4)	0.3833 (4)
Pleuronectes platessa (plaice)	6	0.5176 (2)	0.5095 (1)	0.4798 (0)	0.5382 (0)	n.s.	0.3567 (3)
Pollachius virens (saithe)	3	n.s.	n.s.	n.s.	n.s.	n.s.	n.s.
Scomber scombrus (Atlantic mackerel)	5	n.s.	0.3246 (3)	0.1814 (3)	n.s.	0.0827 (2)	0.0703 (2)
Sprattus sprattus (sprat)	4	n.s.	n.s.	n.s.	n.s.	n.s.	n.s.
Trisopterus esmarkii (Norway pout)	4	0.4610 (4)	n.s.	0.3335 (0)	0.0523 (4)	0.1182 (4)	0.2020 (4)

E^*, optimal embedding dimension of library variable (spatial variability). CV, coefficient of variation; CPUE, catch per unit effort; F, fishing mortality; AMO, Atlantic Multidecadal Oscillation; n.s., none-significant causal effect.

Values in each variable column indicate causal effect of the variable on library variable. Larger values indicate stronger causal effects. Numbers in brackets indicate lag at which causal effect was strongest for the corresponding variable. Causal effect was determined by convergence cross mapping (CCM, see Methods). Yearly data were used in this analysis, because only yearly data were available for fishing mortality. Only significant resutls were shown (p-value < 0.05 in both one-sided Kendall's τ test and Student's t-test on ρ).

Supplementary Table 5 | Causal effect of examined variables, including fishing mortality, on population abundance.

Species	Dimensionality (E^{*})	Library variable: abundance					
		F	CV of CPUE	Age diversity	AMO	Temperature	CV of temperature
Clupea harengus (Atlantic herring)	5	n.s.	n.s.	n.s.	n.s.	n.s.	n.s.
Gadus morhua (Atlantic cod)	2	0.6550 (4)	0.3884 (3)	n.s.	0.1478 (2)	0.3196 (2)	0.0842 (2)
Melanogrammus aeglefinus (haddock)	9	n.s.	n.s.	n.s.	n.s.	n.s.	n.s.
Merlangius merlangus (whiting)	7	0.3646 (1)	0.3398 (2)	0.7028 (0)	0.2785 (2)	0.2411 (0)	0.2167 (4)
Pleuronectes platessa (plaice)	8	n.s.	n.s.	n.s.	n.s.	n.s.	n.s.
Pollachius virens (saithe)	2	0.6075 (0)	0.1047 (1)	0.2096 (1)	0.0656 (0)	0.2853 (1)	n.s.
Scomber scombrus (Atlantic mackerel)	2	n.s.	0.2969 (2)	0.1259 (4)	0.1758 (4)	0.2268 (2)	0.2617 (3)
Sprattus sprattus (sprat)	2	n.s.	0.1468 (4)	n.s.	0.1275 (2)	0.3987 (4)	n.s.
Trisopterus esmarkii (Norway pout)	2	n.s.	0.1592 (4)	0.1299 (1)	0.2628 (2)	0.3254 (0)	n.s.

E^{*}, optimal embedding dimension of library variable (spatial variability). CV, coefficient of variation; CPUE, catch per unit effort; F, fishing mortality; AMO, Atlantic Multidecadal Oscillation; n.s., none-significant causal effect.

Values in each variable column indicate causal effect of the variable on library variable. Larger values indicate stronger causal effects. Numbers in brackets indicate lag at which causal effect was strongest for the corresponding variable. Causal effect was determined by convergence cross mapping (CCM, see Methods). Yearly data were used in this analysis, because only yearly data were available for fishing mortality. Only significant resutls were shown (p-value < 0.05 in both one-sided Kendall's τ test and Student's t-test on ρ).

Supplementary Table 6 | Average over-time influential strengths of selected causal variables, including fishing mortality, on population age diversity.

Species	Library variable: age diversity					
	**F**	**CV of CPUE**	**Abundance**	**AMO**	**Temperature**	**CV of temperature**
Clupea harengus (Atlantic herring)		-0.6692				
Gadus morhua (Atlantic cod)	-0.2519					
Melanogrammus aeglefinus (haddock)				-0.2804		
Merlangius merlangus (whiting)	-0.7208		-0.0486	-0.5416	-0.1068	-0.5221
Pleuronectes platessa (plaice)	0.1537	0.2910	0.1787	-0.4737		-0.0660
Scomber scombrus (Atlantic mackerel)		0.4419	0.0685		-0.2647	-0.0291

F, fishing mortality; CV, coefficient of variation; CPUE, catch per unit effort; AMO, Atlantic Multidecadal Oscillation.

Influential strengths were estimated by S-map model at each time step during study period (see Methods), and were averaged over time and over all significant lagged terms to obtain a more robust result for the short time series (p-value < 0.1, one-sided Student's t-test on ρ). Causal variables were selected according to embedding dimension and their rank of causal effects determined by convergence cross mapping (CCM; see Supplementary Table 4). Yearly data were used in this analysis, because only yearly data were available for fishing mortality.

Supplementary Table 7 | Average over-time influential strengths of selected causal variables, including fishing mortality, on population abundance.

Species	Library variable: abundance					
	F	CV of CPUE	Age diversity	AMO	Temperature	CV of temperature
Gadus morhua (Atlantic cod)	-0.7388					
Merlangius merlangus (whiting)	-0.1586	0.0526	-0.1837	-0.2902	-0.1437	0.4012
Pollachius virens (saithe)	-0.094					
Trisopterus esmarkii (Norway pout)					-0.2776	

F, fishing mortality; CV, coefficient of variation; CPUE, catch per unit effort; AMO, Atlantic Multidecadal Oscillation.

Influential strengths were estimated by S-map model at each time step during study period (see Methods), and were averaged over time and over all significant lagged terms to obtain a more robust result for the short time series (p-value < 0.1, one-sided Student's t-test on ρ). Causal variables were selected according to embedding dimension and their rank of causal effects determined by convergence cross mapping (CCM; see Supplementary Table 5). Yearly data were used in this analysis, because only yearly data were available for fishing mortality.

Supplementary Table 8 | Causal effect of examined variables, including fishing mortality, on population spatial variability.

Species	Dimensionality (*E**)	Library variable: CV of CPUE					
		F	Age diversity	Abundance	AMO	Temperature	CV of temperature
Clupea harengus (Atlantic herring)	3	0.3199 (2)	0.1903 (0)	0.1486 (3)	n.s.	0.3832 (1)	0.2501 (2)
Gadus morhua (Atlantic cod)	4	0.8702 (2)	0.3914 (0)	n.s.	0.4412 (1)	n.s.	n.s.
Melanogrammus aeglefinus (haddock)	4	0.1046 (1)	n.s.	0.0903 (1)	0.3154 (4)	n.s.	n.s.
Merlangius merlangus (whiting)	5	0.4163 (3)	0.0758 (4)	0.3890 (0)	0.4512 (1)	0.1560 (0)	n.s.
Pleuronectes platessa (plaice)	9	n.s.	n.s.	n.s.	n.s.	n.s.	n.s.
Pollachius virens (saithe)	5	n.s.	0.1899 (1)	0.1439 (2)	n.s.	n.s.	0.1369 (1)
Scomber scombrus (Atlantic mackerel)	2	n.s.	n.s.	0.0758 (3)	0.1260 (2)	n.s.	n.s.
Sprattus sprattus (sprat)	5	0.1445 (2)	0.3536 (2)	n.s.	n.s.	0.2462 (1)	0.0842 (3)
Trisopterus esmarkii (Norway pout)	3	n.s.	n.s.	n.s.	n.s.	n.s.	n.s.

E^*, optimal embedding dimension of library variable (spatial variability). CV, coefficient of variation; CPUE, catch per unit effort; F, fishing mortality; AMO, Atlantic Multidecadal Oscillation; n.s., none-significant causal effect.

Values in each variable column indicate causal effect of the variable on library variable. Larger values indicate stronger causal effects. Numbers in brackets indicate lag at which causal effect was strongest for the corresponding variable. Causal effect was determined by convergence cross mapping (CCM, see Methods). Yearly data were used in this analysis, because only yearly data were available for fishing mortality. Only significant resutls were shown (p-value < 0.05 in both one-sided Kendall's τ test and Student's t-test on ρ).

Supplementary Table 9 | Average over-time influential strengths of selected causal variables, including fishing mortality, on population spatial variability.

**Species**	**Library variable: CV of CPUE**						**θ**	**ρ**	***p*-value**
	F	Age diversity	Abundance	AMO	Temperature	CV of temperature			
Gadus morhua (Atlantic cod)	0.0708	-1.1281		-0.7721			1.5	0.6494	< 0.001
Merlangius merlangus (whiting)	-0.455		0.1093	0.4299	-0.2157		8	0.4304	0.0257

CV, coefficient of variation; CPUE, catch per unit effort; F, fishing mortality; AMO, Atlantic Multidecadal Oscillation; θ, nonlinearity of dynamical system; ρ, performance of S-map model.

Influential strengths were estimated by S-map model at each time step during study period (see Methods), and were averaged over time. Causal variables were selected according to embedding dimension and their rank of causal effects determined by convergence cross mapping (CCM; see Supplementary Table 8). Only species with significant S-map results are included (p-value < 0.1, one-sided Student's t-test on ρ). Yearly data were used in this analysis, because only yearly data were available for fishing mortality (F).

Supplementary references

1 Engelhard, G. H., Ellis, J. R., Payne, M. R., ter Hofstede, R. & Pinnegar, J. K. Ecotypes as a concept for exploring responses to climate change in fish assemblages. *ICES J. Mar. Sci.* **68**, 580-591 (2010).
